# Towards a model of biliary atresia - Pilot feasibility study in newborn piglets

**DOI:** 10.1016/j.bbrep.2023.101487

**Published:** 2023-05-23

**Authors:** Thora Wesenberg Helt, Lene Buelund, Lise Borgwardt, Thomas Eriksen, Lars Johansen, Robin de Nijs, Soren Holm, Douglas G. Burrin, Thomas Thymann, Vibeke Brix Christensen

**Affiliations:** a Department of Clinical Physiology and Nuclear Medicine, Rigshospitalet, Copenhagen University Hospital, Denmark; b Department of Veterinary Clinical Sciences, University of Copenhagen, Denmark; c Department of Pediatrics, Rigshospitalet, Copenhagen University Hospital, Denmark; d Childrens Nutrition Research Center, Baylor College of Med, TX, USA; e Department of Veterinary and Animal Science, University of Copenhagen, Denmark

**Keywords:** Biliary atresia, Cholestasis, Liver, Piglets, Animal model, Hepatobiliary scintigraphy

## Abstract

Biliary atresia (BA) is a rare congenital liver disease with unknown etiology, and it is the most common indication for liver transplantation in children. As BA infants suffer from intestinal malabsorption and neurodevelopmental deficits, it is necessary to identify optimal medical and nutritional strategies using appropriate neonatal animal models. We aim to determine the feasibility of using newborn piglets with surgically induced cholestasis (bile duct ligation (BDL)) to mimic clinical features of BA. Six piglets were subjected to abdominal surgery on day 4 after birth. The bile ducts were ligated, and the piglet were followed for up to 12 days. On day 12 the piglets were subjected to a hepatobiliary scintigraphy using the tracer radiolabeled Technetium(99m-tc)-mebrofenin, and blood samples were collected for biochemical profiling. Of the six piglets, hepatobiliary scintigraphy verified that two piglets (BDL) had no excretion of bile into the duodenum, i.e. full cholestasis with a hepatic extraction fraction of 84–87% and clearance time of 230–318 min. One piglet (SHAM) had bile excretion to the duodenum. In accordance with this, the BDL piglets had steatorrhea, and increased levels of bilirubin and gammaglutamyl transferase (GGT). The last three piglets were euthanized due to bile leakage or poor growth. Surgically induced cholestasis in young piglets, may offer an animal model that displays clinical characteristics of biliary atresia, including malabsorption, hyperbilirubinaemia, increased GGT and reduced hepatic excretory function. Following refinement, this animal model may be used to optimize feeding strategies to secure optimal nutrition and neurodevelopment for neonatal cholestasis/BA patients.

## Highlights

• A surgically induced cholestasis in piglets is feasible.
• This animal model displays clinical characteristics of biliary atresia.
• This animal model may be used to determine medical strategies of cholestasis.

## 1 Introduction

Biliary atresia (BA) is a severe and rare congenital liver disease of unknown etiology affecting infants within the first months after birth [1,2]. Although the overall incidence is low, BA is the most common indication for liver transplantation in children [3]. As infants with BA suffer from malabsorption and neurodevelopmental deficits [[4], [5], [6], [7]], there is a need to identify optimal medical or nutritional strategies using appropriate animal models. There is a lack of appropriate animal models of spontaneous development of biliary atresia or fibrous cholangitis. Current animal models can be categorized as 1) surgical bile duct ligation (BDL), 2) experimental viral infections, or 3) toxin induced. In a recent study Garrido et al. induced biliary atresia-like symptoms in rats with induced surgical bile duct ligation [8] and Lainakis et al. suggests that administration of 1,4 phenylene-diisothiocyanate to sows in early pregnancy, induces fibrous cholangitis in the offspring [9]. However, none of the currently available models accurately reflects the clinical condition of biliary atresia seen in infants. This limits the possibilities to explore important aspects of etiology, pathogenesis and treatment. In pigs, the hepatic ductuli form a common hepatic duct which units with the cystic duct from the gall bladder. This bile duct runs to the duodenum via the lesser omentum and enters approximately 3 cm distal to the pylorus similar to human anatomy [10,11]. This anatomy of the bile ducts in pigs allows for surgically induced bile duct ligation, to mimic a condition of cholestasis and clinical features of BA.

As there is a lack of appropriate models of biliary atresia, we propose that surgical bile duct ligation in a piglet model, may offer clinical manifestations of cholestasis that mimics some, but not all, clinical manifestations of biliary atresia. To determine the feasibility of this model, our aim was to do surgical bile duct ligation in a small subsample of pigs, and document postsurgical clinical complications and the degree of either excretion or retention of bile following surgical duct ligation.

## 2 Material and methods

**Fig. 1:**
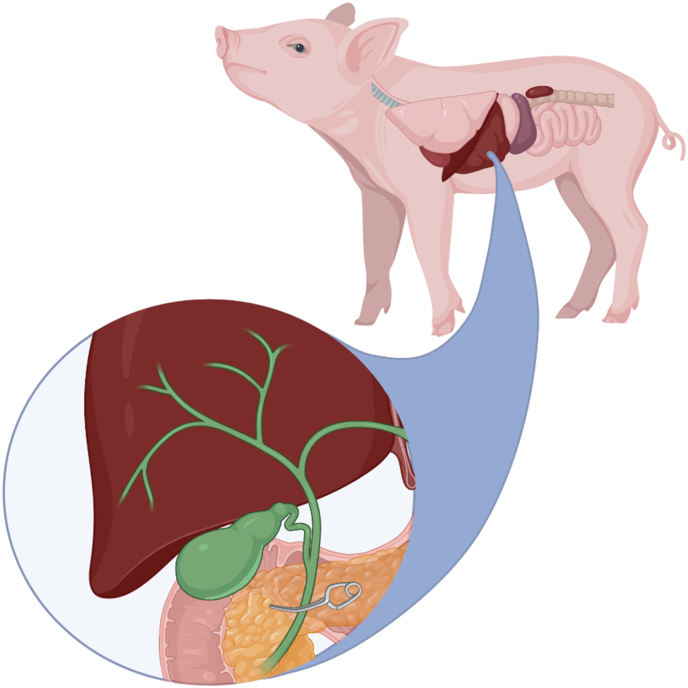
Model of the ligation of the bile ducts.

Six female piglets (Landrace x Yorkshire x Duroc, age 3 days) were purchased from a commercial farm and subjected to abdominal surgery on day 4 after birth. The piglets were transported a very short distance from a commercial farm facility to the experimental unit. Here they were kept individually in tailor-made cages equipped with a heat lamp and access to milk and water Universal anaesthesia (zolazapam, tiletamin, ketamine, butorphanol, and xylazin) and local anaesthesia (lidocaine) were induced, and the bile ducts (ductus cysticus and ductus choledocus) were identified and ligated with a steel Ligaclip (blue size) via a small 2–3 cm ventro-paracostal abdominal incision (Fig. 1). The gall bladder was removed. Following resuscitation, the animals were housed individually and fed a milk replacer (240 ml/kg/day) for up to 12 days. Each piglet was daily weighted and clinically assessed using a score from 1 (best) to 4 (worst) [12]. Assessment criteria included respiratory distress, cyanosis, cold extremities, lethargy, reduced activity, diarrhea, abdominal distension, vomiting, and skin changes. On the last day, a hepatobiliary scintigraphy to determine uptake and clearance of the liver parenchyma (hepatic extraction fraction (HEF) and hepatic clearance rate (HCR)) and leaking after bile duct ligation visualized as excretion to the intestines was performed. Blood tests were taken before euthanization including hematology, liver and kidney parameters, cholesterol, and electrolytes. Animal protocols and procedures were approved by the Danish National Committee on Animal Experimentation and All work was carried out in compliance with the National Guide for the Care and Use of Laboratory Animals.

### 2.1 Hepatobiliary scintigraphy

**Fig. 2:**
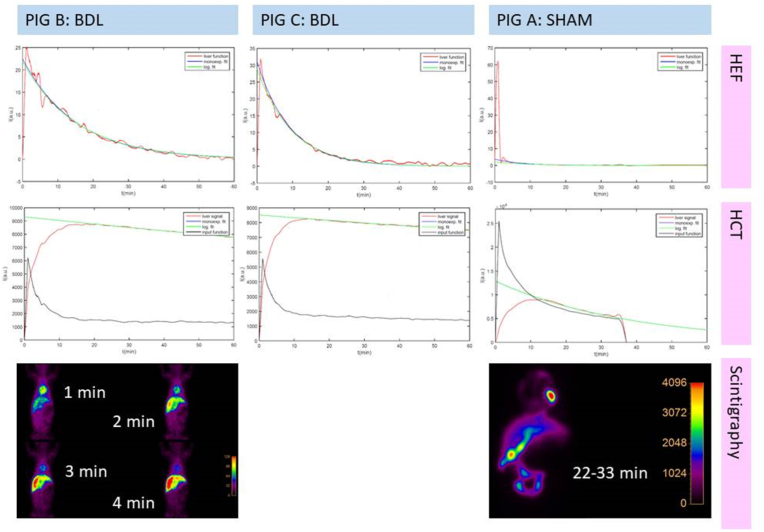
Hepatobiliary scintigraphy with deconvolution analyses in 3 piglets 12 days after BDL attempt.

Piglets were fasted for 2 h before injection with 20 MBq ^99m^Tc-labeled (3-bromo-2,4,6-trimethyl-acetanilide) iminodiacetic acid ([^99m^Tc]Tc-mebrofenin) (Sun Pharmaceutical Industries, Billerica, USA), which was prepared on-site on the day of the scan before injection. After tracer injection, the piglets were placed in the supine position under a Philips triple headed IRIX scanner (Philips Healthcare, Best, Netherlands) equipped with Low Energy High Resolution (LEHR) collimators. Only camera head 2 was utilized in the anterior over patient bed position. A 1-h dynamic planar scanning was performed with 4 min (min) of 1 s frames followed by 56 min with 1 min frames yielding 296 frames in total (Fig. 2). Energy window setting was 140 keV (15% full width). Matrix size was 128 × 128 with an isotropic pixel size of 2.33 mm. The hepatic extraction fraction (HEF), the hepatic clearance time (HCT), blood clearance rate (BClr) and liver clearance rate (LClr) were calculated using deconvolution analysis [[13], [14], [15]]. It was not possible to determine HEF and LClr for piglet A due to limited data acquisition. Regions of interest (ROIs) were manually drawn by the same experienced operator (RN, LB) around the total FOV (indicative of total body activity), the total liver and the heart/large vessels (serving as blood pool) as previously described [13,14]. If no tracer was visualized in the gut, the ligation was sufficient, but if excretion to the intestines were seen the ligation were insufficient verified on autopsy. Autopsy took place 5–6 days after euthanization, because of safety rules in regard to decay of 99mTc-mebrofenin. The piglets were frozen to −18 °C after euthanization and defrosted at 5 °C three days before autopsy. The animal experimental work was approved in 2014 by The Danish Animal Experiments Inspectorate (license number 2014‐15‐0201‐00418).

## 3 Results

**Fig. 3:**
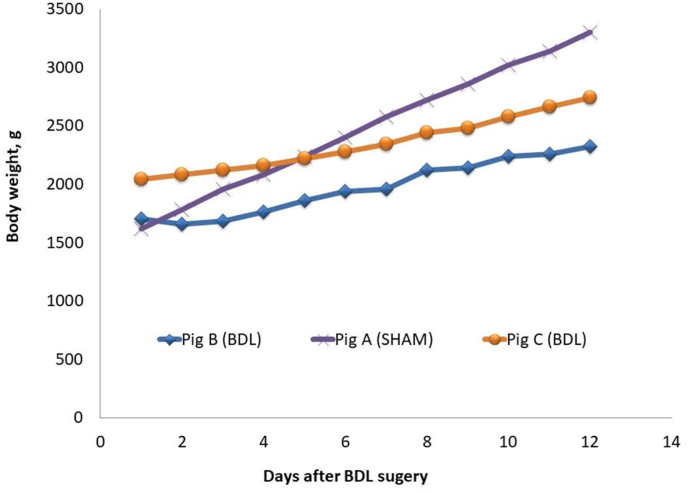
Daily weight of the three piglets after operation.

Out of six piglets, two piglets had leakage of the proximal bile ducts and were euthanized prematurely, and one was euthanized due to poor clinical condition. The remaining three piglets fulfilled the study period. Piglet B and C showed clear signs of steatorrhea and grew 29 and 27 g/kg/day, respectively, whereas piglet A had more solid stool and grew 67 g/kg/day (Fig. 3). Piglet A had unintentionally a continues bile excretion, which at the time of autopsy was found to be due to a misplaced ligature. This piglet was therefore regarded as a sham control. For piglet B and C, the ligature on common bile duct was confirmed at the time of autopsy. These piglets had marked heptic lipidosis and the common bile duct was significantly dilated (20–30 mm), but with no sign of cholascos. Mean clinical score was 1 for all three piglets.

HCT was 225–325 min in piglet B and C, whereas it was only 26 min in piglet A. HEF was 84–93%, LClr was 8.3–10.3%/min and BClr was 13.3–14.9%/min in piglet B and C, whereas HEF, LClr and BClr could not be calculated in piglet A due to unintended partial tracer injection in the heart tissue. Alanine transaminase (ALAT) was 16–20 U/L in piglet B and C, and 15 UL in piglet A. Alkaline phosphatase (BASP) was highest in piglet A at 1388 U/L and 931–1301 U/L in piglet B and C. Gamma-glutamyl transferase (GGT) and bilirubin were highest in piglet B and C at 480–485 U/L and 43–55 μmol/L respectively compared to 31 U/L and 1 μmol/L in piglet A (Table 1).

**Table 1:** Clinical markers, hepatobiliary scintigraphy and blood tests in 3 pigs 12 days after BDL attempt.

		Piglet B (BDL)	Piglet C (BDL)	Piglet A (SHAM)
**Clinical markers**				
Mean growth	g/kg/day	29	27	67
Mean clinical score		1	1	1
**Hepatobiliary scintigraphy**				
HEF#, 1st/2nd analysis	%	83.9/85.6	87.4/92.7	−/−
HCT¶, 1st/2nd analysis	Min	230/225	318/325	26.1/-
BClr§, 1st/2nd analysis	%/min	13.3/14.9	13.7/14.3	−/−
LClr¤, 1st/2nd analysis	%/min	-/10.33	-/8.28	−/−
**Blood tests**				
Hemoglobin	mmol/L	2.9	4.7	3.8
Thrombocytes	10^9/L	2.189	0.978	0.63
ALAT*	U/L	16	20	15
ASAT†	U/L	34	153	38
BASP‡	U/L	1301	931	1388
GGTⅡ	U/L	480	485	31
Total bilirubin	μmol/L	43	55	1
Albumin	g/L	41.86	36.21	28.17
Total protein	g/L	67.97	67.11	40.76
Iron	μmol/L	1.1^1^	1.8	1.2
Phosphate	mmol/L	2.2	2.5	2.7
Calcium	mmol/L	3.11	3.16	2.73
Magnesium	mmol/L	0.89	0.83	0.87
Sodium	mmol/L	129.2	138.6	146.7
Potassium	mmol/L	3.7	3.09	3.95

^1^ *ALAT: Alanine transaminase, †ASAT: Aspartate transaminase, ‡BASP: Alkaline phosphatase, §BClr: Blood clearance rate, ⅡGGT: Gamma-glutamyl transferase, ¶HCT: Hepatic clearance time, #HEF: Hepatic extraction fraction, ¤LClr: Liver clearance rate.

## 4 Discussion

For the two piglets with successful bile duct ligation, clinical and paraclinical data mimics the clinical picture of BA in infants with steatorrhea, reduced growth, and increased bilirubin and GGT indicating cholestasis [1,16]. ALAT was slightly higher in the two BDL piglets and one of the two piglets had increased ASAT indicating some hepatic injury. HEF was only slightly reduced compared to normal, while HCT was vastly increased similar to previous studies looking at bile obstruction [13,14]. Likewise, LClr, but not BCIr, was slightly reduced, which suggest lightly impaired hepatic uptake function as described by Ekman et al. [15]. The last pig without successful ligation had better growth and solid stool, normal bilirubin and GGT [16] and near normal HCT [13,14], but BASP was higher than in the BDL piglets. BClr was reduced, but that can somewhat be explained by the partial tracer in the heart tissue.

The leakiness of bile to the abdominal cavity in two piglets, was presumably related to the steel Ligaclip ligature, which may have traumatized the bile duct in its close proximity. An Absolock might be used instead as they are of polydioxanone (PDS) and may be less traumatizing. Indeed, we have in a subsequent study used soft ligature, and saw no complications due to rupture of the duct [17]. We found that the paracostal incision should not be too small; approximately 3 cm should be sufficient and make it easier to manipulate the pylorus/oral duodenum and visualize the common bile duct. Omitting ligation of the cystic duct will minimize manipulation and oozing hemorrhage from the site of attachment of the gall bladder to the liver. Waiting until the piglets are a few days older before operation might increase survival, but as it is a model of biliary atresia, which is present from birth, the piglets should not be too old before they are operated on. All the piglets had adhesions of the liver to the pylorus region. It could be speculated that such severely dilated (1–2 times normal gall bladder size) common bile duct would be at the same or higher risk of rupture than the gall bladder.

We conclude that surgically induced cholestasis in piglets is feasible and may offer an animal model that displays clinical characteristics of biliary atresia, including malabsorption, hyperbilirubinaemia and reduced hepatic excretory function. Following further refinement, this animal model may be used to determine optimal medical or feeding strategies to secure best possible nutrition on the background of full cholestasis.

## Author contribution

TT, VBC, DB designed the study; TT, VBC, LiB, RN, SH supervised the study; LeB, LJ, TT, TE, VBC, LiB performed the research; RN, TWH analyzed the data; TWH drafted the manuscript. TE, SH, DB gave technical support. All authors have reviewed and approved the manuscript.

## Declaration of competing interest

The authors declare that they have no known competing financial interests or personal relationships that could have appeared to influence the work reported in this paper.

## Data Availability

Data will be made available on request.

## Abbreviations list

[^99m^Tc]Tc-mebrofenin: ^99m^Tc-labeled (3-bromo-2,4,6-trimethyl-acetanilide) iminodiacetic acid
ALAT: Alanine transaminase
ASAT: Aspartate transaminase
BA: Biliary atresia
BASP: Alkaline phosphatase
BClr: Blood clearance rate
BDL: Bile duct ligation
FOV: Field-of-view
GGT: Gamma-glutamyl transferase
HCT: Hepatic clearance time
HEF: Hepatic extraction fraction
LClr: Liver clearance rate
Min: Minutes
PDS: Polydioxanone
ROI: Region of interest
SPECT: Single photon emission computed tomography
